# Permanent draft genome sequence of *Acidiphilium* sp. JA12-A1

**DOI:** 10.1186/s40793-015-0040-y

**Published:** 2015-08-19

**Authors:** Sophie R. Ullrich, Anja Poehlein, Sonja Voget, Michael Hoppert, Rolf Daniel, Andreas Leimbach, Judith S. Tischler, Michael Schlömann, Martin Mühling

**Affiliations:** Institute of Biological Sciences, TU Bergakademie Freiberg, Leipziger Straße 29, 09599 Freiberg, Germany; Genomic and Applied Microbiology & Göttingen Genomics Laboratory, Georg-August-Universität Göttingen, Griesebachstr. 8, 37073 Göttingen, Germany; General Microbiology, Institute of Microbiology and Genetics, Georg-August University of Göttingen, Göttingen, Germany

**Keywords:** *Acidiphilium* sp. JA12-A1, acid mine drainage, AMD, microbial community, acidophilic bacteria

## Abstract

The tenacious association between strains of the heterotrophic alphaproteobacterial genus *Acidiphilium* and chemolithotrophic iron oxidizing bacteria has long been known. In this context the genome of the heterotroph *Acidiphilium* sp. JA12-A1, an isolate from an iron oxidizing mixed culture derived from a pilot plant for bioremediation of acid mine drainage, was determined with the aim to reveal metabolic properties that are fundamental for the syntrophic interaction between *Acidiphilium* sp. JA12-A1 and the co-occurring chemolithoautotrophic iron oxidizer. The genome sequence consists of 4.18 Mbp on 297 contigs and harbors 4015 protein-coding genes and 50 RNA genes. Additionally, the molecular and functional organization of the *Acidiphilium* sp. JA12-A1 draft genome was compared to those of the close relatives *Acidiphilium cryptum* JF-5, *Acidiphilium multivorum* AIU301 and *Acidiphilium* sp. PM DSM 24941. The comparative genome analysis underlines the close relationship between these strains and the highly similar metabolic potential supports the idea that other *Acidiphilium* strains play a similar role in various acid mine drainage communities. Nevertheless, in contrast to other closely related strains *Acidiphilium* sp. JA12-A1 may be able to take up phosphonates as an additional source of phosphor.

**Electronic supplementary material:**

The online version of this article (doi:10.1186/s40793-015-0040-y) contains supplementary material, which is available to authorized users.

## Introduction

Strains of the alphaproteobacterial genus *Acidiphilium* have first been isolated from supposed pure cultures of iron oxidizing bacteria such as *Acidithiobacillus ferrooxidans* [1]. Later on, *Acidiphilium* spp. have also been identified as characteristic members of the microbial communities in acid mine drainage and mining associated water bodies [2–5]. Although the physiological role of these heterotrophic acidophiles within the microbial community has not yet been completely elucidated, the tenacious association between them and the chemolithoautotrophic iron oxidizers has often been reported to be problematic for the isolation of the iron oxidizing bacteria [1, 6, 7]. Several attempts have been undertaken to investigate the interaction between the iron oxidizing bacterium *Acidithiobacillus ferrooxidans* and *Acidiphilium* spp. In a co-culture with *Acidiphilium acidophilum* the increased growth rate and ferrous iron oxidation rate of *Acidithiobacillus ferrooxidans* have indicated a stimulating influence of *Acidiphilium**acidophilus* on *Acidithiobacillus ferrooxidans* [8]. A stable isotope probe based proteome analysis of an *Acidithiobacillus ferrooxidans*/*Acidiphilium cryptum* mixed culture has revealed carbon dioxide transfer from the heterotroph to the iron oxidizing bacterium [9]. Based on the absence of organic carbon and energy sources in the cultivation media of iron oxidizing bacteria it has been suggested that *Acidiphilium* spp. benefit in turn from secreted metabolites and remnants of the biomass from the iron oxidizers by utilizing them as carbon and energy sources [10–12].

Since such an interaction is not only relevant for the isolation and cultivation of iron oxidizing bacteria but also for the general understanding of the ecology of microbial communities in AMD, we were interested in elucidating the potential of *Acidiphilium* for such a syntrophic interaction. Therefore we sequenced and analyzed the genome of *Acidiphilium* sp. JA12-A1 with special focus on transport systems for the uptake of nutrients, the pathways of nutrient assimilation and the general energy metabolism. The resulting permanent draft genome was also compared to the genomes of the close relatives *Acidiphilium cryptum* JF-5, *Acidiphilium multivorum* AUI301 and *Acidiphilium* sp. PM DSM 24941 regarding the genome structure and the functional organization.

### Organism Information

#### Classification and features

Strain *Acidiphilium* sp. JA12-A1 was detected as the heterotrophic contamination in the mixed culture JA12 of a novel chemolithoautotrophic iron oxidizing bacterium [13], which is related to “*Ferrovum myxofaciens**”* P3G [7, 14]. The iron oxidizing mixed culture originated from a pilot plant for the biological remediation of AMD close to a lignite mining site in Lusatia, Germany [5, 13, 15]. *Acidiphilium* sp. JA12-A1 was isolated from the mixed culture by cultivation in SJH medium [16, 17] (Table 1, Additional file 1).

**Table 1:** Classification and general features of *Acidiphilium* sp. JA12-A1 [32]

MIGS ID	Property	Term	Evidence code^a^
	Classification	Domain *Bacteria*	TAS [32]
		Phylum *Proteobacteria*	TAS [33–35]
		Class *Alphaproteobacteria*	TAS [34, 36]
		Order *Rhodospirillales*	TAS [37, 38]
		Family *Acetobacteraceae*	TAS [39, 40]
		Genus *Acidiphilium*	TAS [2, 41, 42]
		Species *Acidiphilium* sp.	TAS [2]
		Strain: JA12-A1	TAS [2]
	Gram stain	Negative	NAS
	Cell shape	Rod	IDA
	Motility	Motile	IDA
	Sporulation	Not reported	
	Temperature range	Mesophile	NAS
	Optimum temperature	30 °C	NAS
	pH range; Optimum	Not reported	
	Carbon source	Heterotroph (galactose, glucose, tryptic soy broth, fructose, yeast extract)	NAS
MIGS-6	Habitat	Acid mine drainage	NAS
MIGS-6.3	Salinity	Not reported	
MIGS-22	Oxygen requirement	Aerobic, anaerobic	NAS
MIGS-15	Biotic relationship	Free-living	NAS
MIGS-14	Pathogenicity	Non-pathogen	NAS
MIGS-4	Geographic location	Lignite mining site, Lusatia, Germany	NAS
MIGS-5	Sample collection	2011	NAS
MIGS-4.1	Latitude	51° 28' 10.38'' N	NAS
MIGS-4.2	Longitude	14° 28' 22.19'' E	NAS
MIGS-4.4	Altitude	125.45 m	NAS

^a^Evidence codes - IDA: Inferred from Direct Assay; TAS: Traceable Author Statement (i.e., a direct report exists in the literature); NAS: Non-traceable Author Statement (i.e., not directly observed for the living, isolated sample, but based on a generally accepted property for the species, or anecdotal evidence). These evidence codes are from the Gene Ontology project [43]

**Fig. 1:**
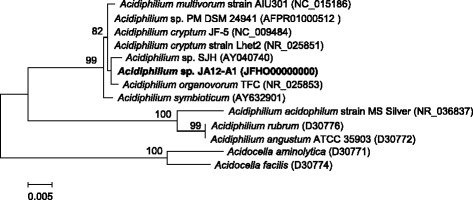
Dendrogram of strains of the genus *Acidiphilium* - based on partial 16S rRNA gene sequences. The dendrogram was calculated with MEGA5 [20] using the Maximum Likelihood method based on the Jukes-Cantor model [21]. The analyzed sequences were aligned by CLUSTALW [22]. The clustering of the sequences was tested by the bootstrap approach with 1000 repeats. The length of the tree branches was scaled according to the number of substitutions per site (see size bar). All strains used in the analysis, except *Acidiphilium cryptum* JF-5 and *Acidiphilium* sp. SJH, are type strains of their respective species [23–30] with *Acidiphilium cryptum* representing the genus *Acidiphilium* as the designated type species [2]. *Acidocella aminolytica* (D300771) and *Acidocella facilis* (D30774) were used as outgroup. The 16S rRNA gene sequence for *Acidiphilium* sp. PM DSM 24941 can be found under the locus tag APM_R0045 on contig Ctg_00688 (AFPR01000512) of the whole genome shotgun sequence. Whole genome sequences are only available for *Acidiphilium cryptum* JF-5, *Acidiphilium multivorum* AIU301, *Acidiphilium* sp. PM DSM 24941 and *Acidiphilium angustum* ATCC 35903 (GOLD project IDs: Gc00559, Gc01862, Gi09776, Gi0051610; accession numbers: NC_009484, NC_015186; AFPR00000000, JNJH00000000)

The complete 16S rRNA gene sequence of *Acidiphilium* sp. JA12-A1 was compared to the non-redundant nucleotide collection of the NCBI using NCBI MegaBLAST [18, 19]. The analysis of the 100 best hits revealed a sequence similarity of 99 % to 16S rRNA gene fragments of *Acidiphilium multivorum* AUI301, *Acidiphilium cryptum* JF-5, *Acidiphilium organovorum* TFC, *Acidiphilium* sp. SJH, and “*Acidiphilium**symbioticum”* and others, and a sequence similarity of 95 % to *Acidiphilium acidophilum* MS Silver, *Acidiphilium angustum* ATCC 35903 and *Acidiphilium rubrum*. These gene fragments also formed the basis for the calculation of a dendrogram illustrating the phylogenetic neighborhood of *Acidiphilium* sp. JA12-A1 (Fig. 1).

The 16S rRNA gene sequences cluster into two distinct subgroups within the genus *Acidiphilium*. The novel strain *Acidiphilium* sp. JA12-A1 belongs to the same subgroup as *Acidiphilium cryptum**JF-5,**Acidiphilium multivorum* AIU301 and *Acidiphilium* sp. PM DSM 24941.

**Fig. 2:**
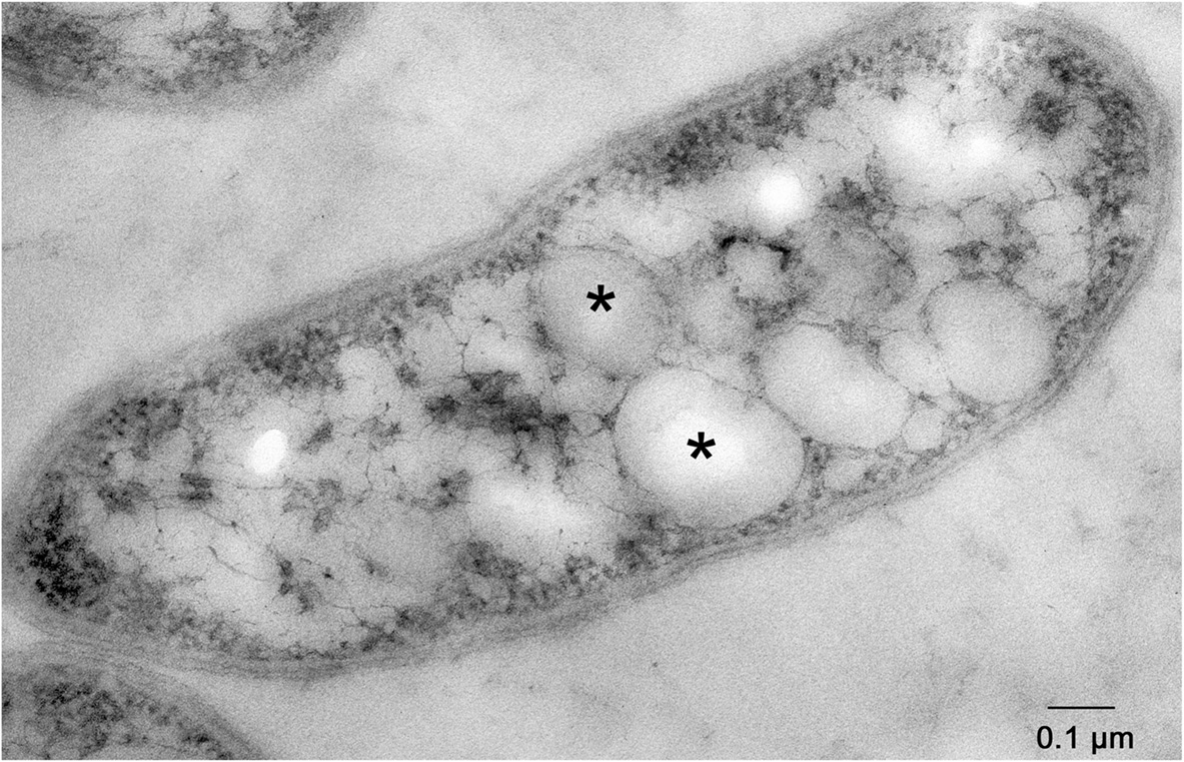
Transmission electron micrograph of *Acidiphilium* sp. JA12-A1 (ultrathin section, post-staining with 4 % uranyl acetate). PHB granula are marked by asterisks. The cells were harvested at the beginning of the fast growth phase

In terms of physiological features *Acidiphilium* sp. JA12-A1 appears to be closely related to the type strain *Acidiphilium cryptum* Lhet2 [2]: *Acidiphilium* sp. JA12-A1 is a Gram-negative, rod-shaped (ca. 1.9 μm × 0.7 μm), motile alphaproteobacterium, which lives under acidophilic conditions. It has a chemoorganotrophic lifestyle growing with galactose, fructose, yeast extract and soy broth as growth substrates. In the mixed culture with the iron oxidizer “*Ferrovum**”* sp. JA12 [31] the proportion of *Acidiphilium* sp. JA12-A1 was estimated by terminal restriction fragment length polymorphism (T-RFLP) analysis to vary between 1 % and 50 % depending on the ferrous iron concentration and growth phase (unpublished results). An electron micrograph of *Acidiphilium* sp. JA12 is provided in Fig. 2.

## Genome sequencing information

### Genome project history

The genome of *Acidiphilium* sp. JA12-A1 was sequenced to obtain genetic information on physiological properties that may play a fundamental role in its tenacious association with the co-occurring iron oxidizing bacterium in the mixed culture JA12. The permanent draft genome sequence is available at the NCBI with the accession number JFHO00000000 (genome project number 238988). The cultivation and genome sequence analysis was undertaken at the TU Bergakademie Freiberg while the genome sequencing and annotation was performed at Göttingen Genomics Laboratory (G_2_L). Table 2 provides a summary of the project information according to MIGS compliance [32].

**Table 2:** Project information

MIGS ID	Property	Term
MIGS 31	Finishing quality	Improved high-quality draft
MIGS-28	Libraries used	Two genomic libraries: 454 pyrosequencing shotgun library, Illumina paired-end library (1 kb insert size)
MIGS 29	Sequencing platforms	454 GS FLX Titanium, Illumina GAII
MIGS 31.2	Fold coverage	18.7 × 454, 54.8 × Illumina
MIGS 30	Assemblers	Newbler 2.8, MIRA 3.4
MIGS 32	Gene calling method	YACOP, Glimmer
	Locus Tag	ACIDI
	Genbank ID	JFHO01000000
	GenBank Date of Release	2014-05-20
	GOLD ID	Gi0008223
	BIOPROJECT	PRJNA238988
MIGS 13	Source Material Identifier	TU BAF Acidi
	Project relevance	Environmental and biotechnological

### Growth conditions and genomic DNA preparation

*Acidiphilium* sp. JA12-A1 was cultivated in liquid SJH medium [16, 17] at 30 °C. It was continuously shaken on a rotary shaker at 120 rpm. The cells were harvested by centrifugation at 10,000 × g. The DNA was isolated using the Ultra Clean™ Microbial DNA Isolation Kit (MoBio, Carlsbad, CA) according to the manufacturer’s instructions.

### Genome sequencing and assembly

Genome sequencing of *Acidiphilium* sp. JA12-A1 was performed via a hybrid approach using the 454 GS-FLX TitaniumXL system (Titanium GS70 chemistry, Roche Life Science, Mannheim, Germany) and the Genome Analyzer II (Illumina, San Diego, CA). Shotgun libraries were prepared according to the manufacturer's protocols, resulting in 126,343 reads for 454 shotgun and 10,136,209 112-bp paired-end Illumina reads. We used all 126.343 454 shotgun reads and 3,000,000 of the 112-bp paired-end Illumina reads for the initial hybrid *de-novo* assembly, which was calculated using the MIRA 3.4 [44] and Newbler 2.8 (Roche Life Science, Mannheim, Germany) software. The final assembly contained 297 contigs with a 73.5-times coverage on average.

### Genome annotation

The software tools YACOP and Glimmer [45] were used for automatic gene prediction, while identification of rRNA and tRNA genes was performed using RNAmmer and tRNAscan, respectively [46, 47]. An automatic annotation was performed within the integrated microbial genomes-expert review (IMG-ER) system [48, 49] and subsequently curated manually by using the Swiss-Prot, TrEMBL, and InterPro databases [50].

## Genome Properties

The draft genome of *Acidiphilium* sp. JA12-A1 consists of 4.18 Mbp on 298 contigs, of which 99 have a length of at least 10 kbp. Genome features are summarized in Table 3. The average G + C content is 66.9 %. The draft genome encodes 4065 genes in total, of which 4015 (98.8 %) are predicted protein coding genes and 50 (1.2 %) are RNA genes. 2663 (65.5 %) genes are assigned to COG groups (Table 4), 1238 (30.5 %) are connected to KEGG pathways and 520 (12.8 %) are assigned to the transporter classification. A comparison of genome features of *Acidiphilium* sp. JA12-A1 to the genomes of *Acidiphilium cryptum* JF-5, *Acidiphilium multivorum* AUI301 and *Acidiphilium* sp. PM, DSM 24941 is provided in Table 5.

**Table 3:** Genome statistics *Acidiphilium* sp. JA12-A1

Attribute	Value	% of Total
Genome size (bp)	4,184,331	100.0
DNA coding (bp)	3,699,946	88.4
DNA G + C (bp)	2,801,106	66.9
DNA scaffolds	298	
Total genes	4,065	100.0
Protein coding genes	4,015	98.8
RNA genes	50	1.2
Pseudo genes	293	7.2
Genes in internal clusters	3,092	76.1
Genes with function prediction	3,193	78.6
Genes assigned to COGs	2,663	65.5
Genes with Pfam domains	3,191	78.5
Genes with signal peptides	268	6.6
Genes with transmembrane helices	857	21.1
CRISPR repeats	Not reported

**Table 4:** Number of genes associated with general COG functional categories

Code	Value	% age	Description
J	147	5.0	Translation, ribosomal structure and biogenesis
A	0	0.0	RNA processing and modification
K	180	6.1	Transcription
L	157	5.3	Replication, recombination and repair
B	2	0.1	Chromatin structure and dynamics
D	167	5.7	Cell cycle control, Cell division, chromosome partitioning
V	35	1.2	Defense mechanisms
T	77	2.6	Signal transduction mechanisms
M	167	5.7	Cell wall/membrane biogenesis
N	44	1.5	Cell motility
U	77	2.6	Intracellular trafficking and secretion
O	107	3.6	Posttranslational modification, protein turnover, chaperones
C	260	8.8	Energy production and conversion
G	247	8.3	Carbohydrate transport and metabolism
E	294	10.0	Amino acid transport and metabolism
F	66	2.2	Nucleotide transport and metabolism
H	125	4.2	Coenzyme transport and metabolism
I	164	5.6	Lipid transport and metabolism
P	124	4.2	Inorganic ion transport and metabolism
Q	89	3.0	Secondary metabolites biosynthesis, transport and catabolism
R	320	10.8	General function prediction only
S	241	8.2	Function unknown
-	1,400	34.4	Not in COGs

The total is based on the total number of protein coding genes in the genome

**Table 5:** Comparison of genome features of *Acidiphilium* sp. JA12-A1 to close relatives

Genome features	Genome name			
	*A. cryptum* JF-5^a^	*A. multivorum* AIU301^b^	*Acidiphilium* sp. PM DSM 24941^c^	*Acidiphilium* sp. JA12-A1^d^
Sequencing status	Finished	Finished	Draft	Permanent draft
Genome size (Mbp)	4.0	4.2	3.9	4.2
Number of plasmids	8	8	9	Not reported
GC (percentage)	67.1 %	67.0 %	66.4 %	66.9 %
Total gene count	3,701	4,004	3,908	4,065
Number of CDS genes (percentage)	3,637 (98.3 %)	3,948 (98. 6 %)	3,859 (98.8 %)	4,015 (98.8 %)
Number of RNA genes	64 (1.7 %)	56 (1.4 %)	49 (1.3 %)	50 (1.2 %)
Number of genes assigned to COGs (percentage)	2,830 (79.1 %)	3,188 (76.5 %)	3,116 (79.7 %)	2,663 (65.5 %)
Number of genes connected to KEGG pathways (percentage)	1,197 (32.3 %)	1,283 (32.0 %)	1,133 (29.0 %)	1,238 (30.5 %)
Number of genes assigned to enzymes (percentage)	1,055 (28.5 %)	1,107 (27.7 %)	965 (24.7 %)	1,076 (26.5 %)
Number of genes assigned to transporter classification (percentage)	524 (14.1 %)	562 (14.0 %)	573 (14.7 %)	520 (12.8 %)
Number of genes coding transmembrane proteins (percentage)	817 (22.1 %)	880 (22.0 %)	839 (21.5 %)	857 (21.1 %)
Number of genes with signal peptides (percentage)	240 (6.5 %)	266 (6.6 %)	232 (5.9 %)	268 (6.6 %)

^a^accession number: NC_009484; ^b^NC_015186; ^c^AFPR00000000; ^d^JFHO00000000

## Insights from the genome sequence

In order to understand the potential interaction between *Acidiphilium* sp. JA12-A1 and the iron oxidizer “*Ferrovum**”* sp. JA12 in the mixed culture we analyzed the genome of *Acidiphilium* sp. JA12-A1 with special focus on genes that may be involved in the utilization of “*Ferrovum**”* derived organic substances as an energy source and as growth substrates.

The genome analysis revealed six genes that encode for putative oligo- and polysaccharide hydrolyzing enzymes, among which we identified α-amylases or amylase-related enzymes, β-glucosidase, endoglucanase, a trehalase and a glycogen-debranching enzyme. *Acidiphilium* sp. JA12-A1 may use these enzymes to break down polysaccharides that are part of the cell envelope of the iron oxidizer “*Ferrovum**”* or that are excreted as slimes. Applying the EBI InterProScan to the sequences of these enzymes resulted in predicted N-terminal signal peptides in the β-glucosidase and endoglucanase which indicates a potential excretion of these enzymes.

The genome of *Acidiphilium* sp. JA12-A1 encodes a variety of transport systems to take up secreted organic compounds or the products of the hydrolysis of polysaccharides. These transport systems comprise annotated sugar transporters or sugar phosphate permeases of the major facilitator family, 15 ABC-transport systems for mono- and disaccharides and a phosphotransferase system (PTS) of the fructose type. The ABC-transporters are predicted to take up ribose, xylose, galactose or similar monosaccharides. The PTS in *Acidiphilium* sp. JA12-A1 consists, similar to the PTS of other *Acidiphilium* strains, of two fusion proteins (HPr/EI/EIIA and EIIB/EIIC).

Based on the genome sequence we reconstructed the metabolic pathways that may enable *Acidiphilium* sp. JA12-A1 to gain energy by the complete aerobic oxidation of organic compounds, preferably of monosaccharides. Although we did not identify the fructose-6-phosphate kinase, one of the key enzymes of the glycolysis, *Acidiphilium* sp. JA12-A1 may bypass the reaction *via* the activity of enzymes of the pentosephosphate pathway, thus still being able to convert glucose to acetyl-CoA. Acetyl-CoA is further oxidized to carbon dioxide by the citrate cycle and the electrons are transferred to oxygen by the protein complexes of the aerobic respiratory chain. We also identified gene clusters encoding the subunits of a photosynthetic reaction center, associated cytochromes and proteins involved in the biogenesis of the reaction center proteins that may enable *Acidiphilium* sp. JA12-A1 to use light as additional energy source.

In addition to the aerobic respiration *Acidiphilium* sp. JA12-A1 may also be able to reduce ferric iron under microaerobic or anaerobic conditions as it has been described for other *Acidiphilium* strains [51, 52]. Despite of the experimental evidence for the ferric iron reduction, the proteins that are involved in the direct reduction of ferric iron in acidophiles have still not been identified [53]. The genome analysis of *Acidiphilium* sp. JA12-A1 also failed to reveal any further details of the electron transfer processes to ferric iron.

Apart from providing the source of energy the sugar compounds also appear to be the preferred carbon source for the biomass production in *Acidiphilium* sp. JA12-A1. We inferred the pathways that are necessary for the conversion of the monosaccharides to the precursors of the biomass production, such as the amino sugar and nucleotide sugar metabolism, the citrate cycle, the fatty acid synthesis and the purine and pyrimidine metabolism. Besides the synthesis of biomass there is genetic evidence for the storage of carbon compounds as polyhydroxybutyrate (PHB) which is further supported by transmission electron microscopic analysis of representative cells showing PHB granula (Fig. 2). *Acidiphilium* sp. JA12-A1 also appears to be able to fix carbon dioxide heterotrophically, since its genome encodes a pyruvate carboxylase and a pyruvate carboxykinase.

### Extended insights

**Fig. 3:**
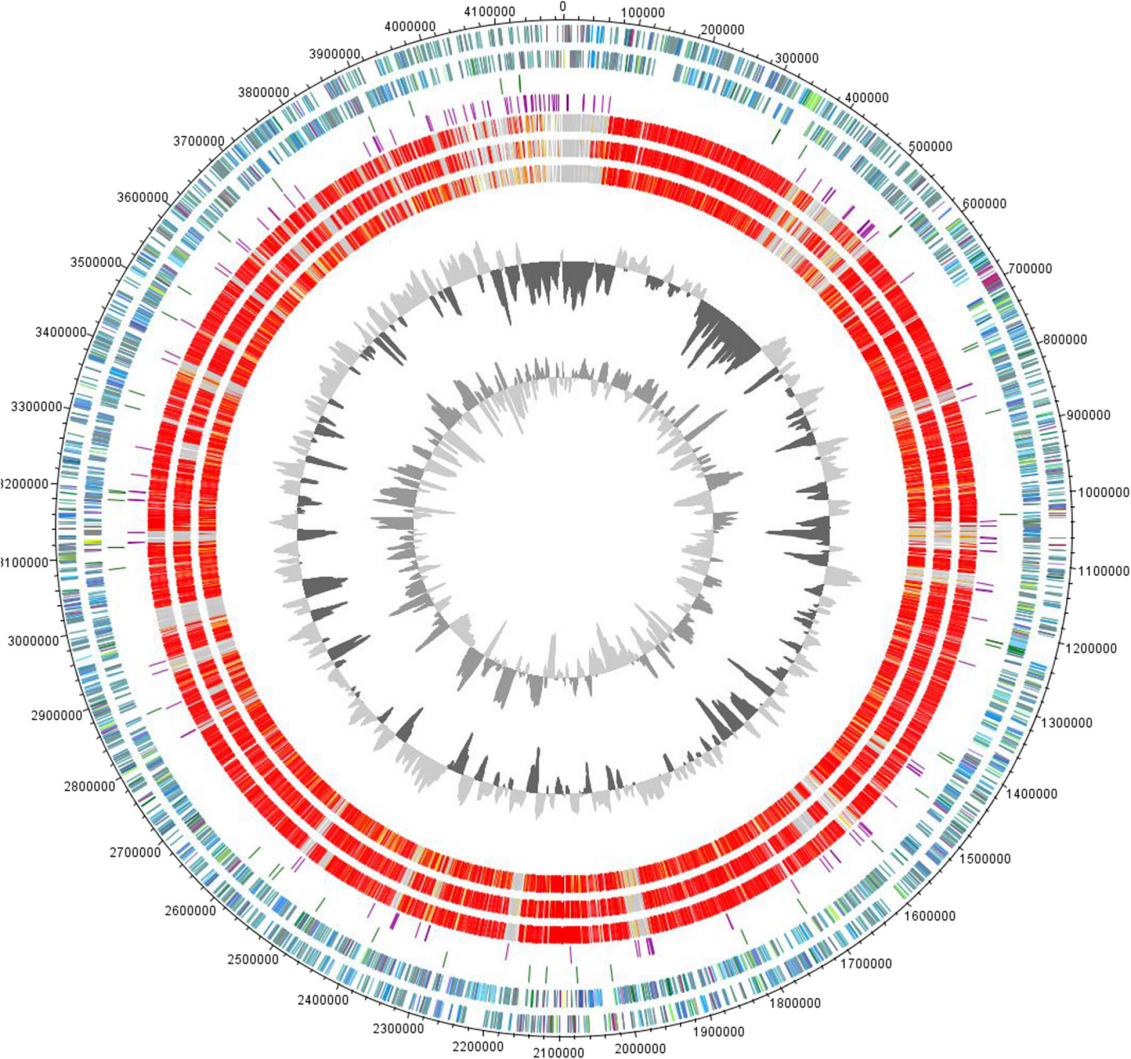
Circular representation of the genome comparison of *Acidiphilium* sp. JA12-A1 with other *Acidiphilium* strains. A: The genes encoded by the leading and the lagging strand (outer circles 1 and 2) of *Acidiphilium* sp. JA12-A1 are marked in COG colors in the artificial chromosome map. The genes for tRNAs and transposases in *Acidiphilium* sp. JA12-A1 are shown in circles 3 and 4, respectively. The presence of orthologous genes is indicated for the genomes of *Acidiphilium cryptum* JF-5 (CP000689-CP000697), *Acidiphilium multivorum* AIU301 (AP012035-AP012043) and *Acidiphilium* sp. PM DSM 24941 (circle 5 to 7). The two innermost plots represent the GC-content and the GC-skew

**Fig. 4:**
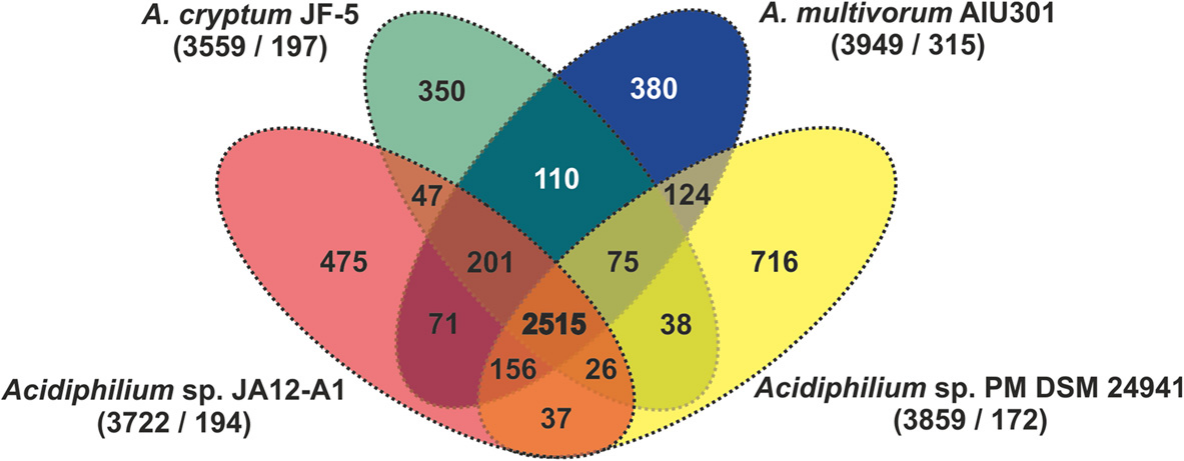
Venn diagramm of the genome comparison of *Acidiphilium* sp. JA12-A1 with other *Acidiphilium* strains. Venn diagram showing the orthologous genes between *Acidiphilium* sp. JA12-A1, *Acidiphilium cryptum* JF-5 (CP000689-CP000697), *Acidiphilium multivorum* AIU301 (AP012035-AP012043) and *Acidiphilium* sp. PM DSM 24941 (AFPR00000000). Ortholog detection was done with the Proteinortho software (blastp) with an similarity cutoff of 50 % and an E-value of 1e-10. The total number of genes and paralogs, respectively, are depicted under the corresponding species name. Open reading frames (ORFs) that were classified as pseudogenes, were not included in this analysis

Although there are four genome sequences of species belonging to the genus *Acidiphilium* to compare the genome of strain JA12-A1 with, we focused our comparative genomics approach on *Acidiphilium cryptum* JF-5, *Acidiphilium multivorum* AUI301, *Acidiphilium* sp. PM DSM 24941 and *Acidiphilium* sp. JA12-A1. A comparison of the genomes of *Acidiphilium* sp. JA12-A1 and *Acidiphilium angustum* ATCC 35903 confirmed the phylogenetic distance and revealed that these genomes cannot be meaningfully aligned (results not shown). Therefore, the circular representation of the genome comparisons (Fig. 3) and the Venn diagram summarizing orthologous genes between the genomes are limited to strains belonging to the same phylogenetic cluster as *Acidiphilium* sp. JA12-A1 (Fig. 4).

The circular representation of genome sequences of four *Acidiphilium* strains revealed a high structural similarity of the genomes (Fig. 3). To identify orthologous genes between all four organisms, we performed a whole genome comparison. To prepare the data for analysis we used the scripts ncbi_ftp_download v0.2, cat_seq v0.1 and cds_extractor v0.6 [54] and Proteinortho v5.04 [55] with a similarity cutoff of 50 % and an E-value of 1e-10. Paralogous genes detected for all genomes were not included into this approach. All four strains have a core genome comprising 2515 genes, which is up to 70 % of the genes present in a single genome (Fig. 4). *Acidiphilium* JA12-A1 has 2943 orthologous genes in common with *Acidiphilium multivorum* AIU301, 2789 with *Acidiphilium cryptum* JF-5 and 2734 with *Acidiphilium* sp. PM DSM 24941. We detected the highest number of orthologous genes (2901) between *Acidiphilium cryptum* JF-5 and *Acidiphilium multivorum* AIU301. *Acidiphilium* sp. PM DSM 24941 and *Acidiphilium multivorum* AIU301 have 2870 in common, while *Acidiphilium cryptum* JF-5 and *Acidiphilium* sp. PM DSM 24941 share 2654 genes. *Acidiphilium* sp. PM DSM 24941 harbors the highest number of singletons (716) followed by *Acidiphilium* JA12-A1 with 475, *Acidiphilium multivorum* AIU301 with 381 and *Acidiphilium cryptum* JF-5 with 350, respectively. This, therefore, confirms the high degree of similarity among the various *Acidiphilium* strains as already concluded from the 16S rRNA gene based phylogeny (Fig. 1). Moreover, the high degree of congruence of the selected genome features provided in Table 5 demonstrates the high similarity among the four genomes with respect to the functional organization, (e.g. number of genes assigned to various COG functional categories (not shown), and pathways of the central metabolism).

Despite the high similarity in genome organization and content there are also unique genes in each of the *Acidiphilium* species that were included in this comparative genome analysis. For instance, *Acidiphilium* sp. JA12-A1, *Acidiphilium cryptum* JF-5 and *Acidiphilium multivorum* AUI301 contain a cluster of homologous genes encoding phosphonate C-P-lyases which are required for utilization of organic phosphate compounds. However, of those only *Acidiphilium* sp. JA12-A1 encodes a putative phosphonate specific ABC transporter. ABC transporter encoding genes are usually clustered. In the case of *Acidiphilium* sp. JA12 the genes are spread within the genome indicating that these have possibly been acquired *via* horizontal gene transfer.

## Conclusions

The microbial communities of AMD and mining associated water bodies have been investigated in some detail over the last decades [3, 5, 10–12, 14, 56–58]. All of these reports agree on the supposed role of heterotrophic microorganisms, including members of the genus *Acidiphilium*, regarding their utilization of organic substances secreted by other community members or derived from microbial cell decay.

Analyzing the genome sequence of the novel strain *Acidiphilium* sp. JA12-A1 we inferred such an interspecies carbon transfer in an iron oxidizing mixed culture derived from a pilot plant for the biological remediation of AMD. The potential carbon transfer involves *Acidiphilium* sp. JA12-A1 excreting polysaccharide hydrolyzing enzymes, such as β-glucosidases or endoglucanases, to break down cell envelope polysaccharides from decaying cells and from the co-occurring iron oxidizer that is related to *F. myxofaciens* P3G [7]. Monosaccharides originating from polysaccharide hydrolysis or from lysed cells are taken up by *Acidiphilium* sp. JA12-A1 *via* specific uptake systems to produce bacterial biomass. Alternatively, the monosaccharides or parts thereof are oxidized to gain energy for the cellular metabolism. Under aerobic conditions the electron donor is completely oxidized to carbon dioxide which is the preferred carbon source for the autotrophic iron oxidizer. However, the iron oxidizer may not only profit from the local increase of the carbon dioxide availability but also from the removal of organic compounds by *Acidiphilium* sp. JA12-A1, since chemolithoautotrophic iron oxidizers have long been known to be sensitive to organic compounds [59]. The sum of these potential interactions may account for the tenacious association of both organisms in the mixed culture and provide an explanation for the difficulties encountered when attempting to obtain pure cultures of the iron oxidizing bacteria.

In order to experimentally substantiate such an interspecies carbon transfer we suggest to analyze, similar to the study of Kermer et al. [9], secreted metabolites in combination with a stable isotope approach (^13^C-labelled carbon dioxide) since this may reveal the actual metabolites that are utilized by *Acidiphilium* sp. JA12-A1 in the mixed culture. This approach may not only extend our knowledge of the proposed interspecies carbon transfer [9], but also elucidate whether *Acidiphilium* sp. JA12-A1 incorporates carbon dioxide heterotrophically by carboxylation reactions under the conditions provided within the mixed culture. In *Acidiphilium rubrum* the incorporation of carbon dioxide was described to be enhanced under aerobic-light conditions with the required energy provided by light utilization *via* a photosynthetic reaction center and phototrophic pigments [60]. We identified gene clusters homologous to those described for *Acidiphilium rubrum* and other *Acidiphilium* strains in the genome of *Acidiphilium* sp. JA12-A1 hinting at a potential photosynthetic activity. However, since none of the described *Acidiphilium* strains seems to be capable of using light as sole source of energy [61], it has been proposed that the photosynthetic activity is used to pump protons across the cytoplasmic membrane in order to stabilize the proton balance between the acidic environment and the neutral cytoplasm [60].

*Acidiphilium* strains are also thought to play a direct role in the iron cycle by regenerating dissolved ferrous iron through the reduction of ferric iron under microaerobic and anoxic conditions [11, 62]. Other studies have shown that ferrous iron is regenerated from the reduction of ferric iron minerals by *Acidiphilium* spp. and other acidophilic ferric iron reducers [52]. The ferrous iron is then available as an energy source for the iron oxidizers again. Details of the pathway of ferric iron reduction could, however, not be deduced from the genome of *Acidiphilium* sp. JA12-A1.

The *Acidiphilium* strains *Acidiphilium cryptum* JF-5, *Acidiphilium multivorum* AUI301, *Acidiphilium* sp. PM DSM 24941 and *Acidiphilium* sp. JA12-A1, which all belong to the same phylogenetic subgroup within the genus *Acidiphilium*, show high similarities regarding their structural and functional genome organization. Since they also share important metabolic traits with respect to growth conditions and nutrient requirements the proposed interaction between *Acidiphilium* sp. JA12-A1 and the iron oxidizer *Ferrovum* spp. may also be true for other members of the genus *Acidiphilium* in their natural habitats.

## Additional file

Additional file 1: Table S1. Associated MIGS record. (DOC 73 kb)

## Abbreviations

AMD: acid mine drainage
PHB: polyhydroxybutyrate
