# Supplementary material for: Permanent draft genome sequence of Acidiphilium sp. JA12-A1
Source: Stand Genomic Sci. 2015 Aug 19;10:56. doi: 10.1186/s40793-015-0040-y (PMC4571130; doi:10.1186/s40793-015-0040-y)
Supplement: Additional file 1: Table S1. — Associated MIGS record. (DOC 73 kb) [file 40793_2015_40_MOESM1_ESM.doc]

**Appendix: Associated MIGS Record**

***Table S1.*** *Associated MIGS record*

| **MIGS-ID** | field name | description |
| --- | --- | --- |
| **MIGS-1** | Submit to INSDC/Trace archives |  |
| **MIGS-1.1** | PID | 238988 |
| **MIGS-1.2** | Trace Archive | SRP043704 |
| **MIGS-2** | MIGS CHECK LIST TYPE | 2.0 |
| **MIGS-3** | Project Name | *Acidiphilium* JA12-A1 |
| **MIGS-4** | Geographic Location | lignite mining site, Lusatia, Germany |
| **MIGS-4.1** | Latitude | 51° 28' 10.38'' N |
| **MIGS-4.2** | Longitude | 14° 28' 22.19'' E |
| **MIGS-4.3** | Depth | not reported |
| **MIGS-4.4** | Altitude | 125.45 m |
| **MIGS-5** | Time of Sample collection | 2011 |
| **MIGS-6** | Habitat (EnvO) | acid mine drainage |
| **MIGS-6.1** | temperature | not reported |
| **MIGS-6.2** | pH | not reported |
| **MIGS-6.3** | salinity | not reported |
| **MIGS-6.4** | chlorophyll | not reported |
| **MIGS-6.5** | conductivity | not reported |
|
| **MIGS-6.6** | light intensity | not reported |
| **MIGS-6.7** | dissolved organic carbon (DOC) | not reported |
| **MIGS-6.8** | current | not reported |
| **MIGS-6.9** | atmospheric data | not reported |
| **MIGS-6.10** | density | not reported |
| **MIGS-6.11** | alkalinity | not reported |
| **MIGS-6.12** | dissolved oxygen | not reported |
| **MIGS-6.13** | particulate organic carbon (POC) | not reported |
| **MIGS-6.14** | phosphate | 0.01 mM |
| **MIGS-6.15** | nitrate | not reported |
| **MIGS-6.16** | sulfates | not reported |
| **MIGS-6.17** | sulfides | not reported |
| **MIGS-6.18** | primary production | not reported |
| **MIGS-7** | Subspecific genetic lineage | not reported |
| **MIGS-9** | Number of replicons | not reported |
| **MIGS-10** | Extrachromosomal elements | not reported |
| **MIGS-11** | Estimated Size | 1 – 1.5 µm |
| **MIGS-12** | Reference for biomaterial or Genome report | not reported |
| **MIGS-13** | Source material identifiers | not reported |
| **MIGS-14** | Known Pathogenicity | none |
|
| **MIGS-15** | Biotic Relationship | free living |
| **MIGS-16** | Specific Host | none |
| **MIGS-17** | Host specificity or range (taxid) | not reported |
| **MIGS-18** | Health status of Host | not reported |
| **MIGS-19** | Trophic Level | heterotroph, decomposer |
| **MIGS-22** | Relationship to Oxygen | aerobic, microaerophilic, anaerobic |
| **MIGS-23** | Isolation and Growth conditions | SJH medium [16, 17] 30 °C, rotary shaker |
| **MIGS-27** | Nucleic acid preparation | Ultra CleanTM Microbial DNA Isolation Kit (MoBio, Carlsbad, California) |
| **MIGS-28** | Library construction | 454 pyrosequencing shotgun library (according to Roche protocol), Illumina paired-end library |
| **MIGS-28.1** | Library size | 454 pyrosequencing shotgun library: 126,343 reads,Illumina paired-end library: 10,136,209 reads Illumina: 1 kb |
| **MIGS-28.2** | Number of reads | 454 pyrosequencing shotgun library: 126,343; Illumina paired-end library: 3,000,000 |
| **MIGS-28.3** | vector | not reported |
| **MIGS-29** | Sequencing method | 454 GS FLX Titanium, Illumina GAII |
| **MIGS-30** | Assembly |  |
| **MIGS-30.1** | Assembly method | Newbler 2.8, MIRA 3.4 |
| **MIGS-30.2** | estimated error rate | not reported |
| **MIGS-30.3** | method of calculation | not reported |
| **MIGS-31** | Finishing strategy |  |
| **MIGS-31.1** | Status | 3: Improved high qualitity draft |
| **MIGS-31.2** | coverage | 18.7 × 454, 54.8 × Illumina |
| **MIGS-31.3** | contigs | 297 |
| **MIGS-32** | Relevant SOPs |  |
|  | Gene calling method | YACOP, Glimmer |
|  | INSDC ID | JFHO00000000 |
|  | NCBI project ID | 238988 |
|  | Database: IMG | 2556793025 |
|  | Genbank Date of Release | 2014-05-20 |
|  | GOLD ID | Gi0008223 |
|  | Project relevance | Environmental and biotechnological |
| **MIGS-33** | Relevant e-resources | Data, GenBank (http://www.ncbi.nlm.nih.gov/nuccore/636536390; http://www.ncbi.nlm.nih.gov/sra?Db=sra&DbFrom=bioproject&Cmd=Link&LinkName=bioproject_sra&LinkReadableName=SRA&ordinalpos=1&IdsFromResult=238988), DOE https://img.jgi.doe.gov/cgi-bin/er/main.cgi?section=TaxonDetail&page=taxonDetail&taxon_oid=2571042905). Seq Center, Goettingen Genomics Laboratory (http://appmibio.uni-goettingen.de/) |
